# The Important Role of Aquaglyceroporin 7 in Health and Disease

**DOI:** 10.3390/biom14101228

**Published:** 2024-09-28

**Authors:** Jing Liu, Ziwei Xia, Shuhong Peng, Juanjuan Xia, Ruixiang Xu, Xin Wang, Fei Li, Weifeng Zhu

**Affiliations:** 1 School of Pharmacy, Jiangxi University of Chinese Medicine, Nanchang 330004, China; 20111014@jxutcm.edu.cn (J.L.); xiaziwei@jxutcm.edu.cn (Z.X.); xiajuanjuan@jxutcm.edu.cn (J.X.); xuruixiang1@jxutcm.edu.cn (R.X.); wangxin76@jxutcm.edu.cn (X.W.); 2 Research Center for Differentiation and Development of Traditional Chinese Medicine Basic Theory, Jiangxi University of Chinese Medicine, Nanchang 330004, China; 20030770@jxutcm.edu.cn; 3 Frontiers Science Center for Disease-Related Molecular Network, West China Hospital, Sichuan University, Chengdu 610041, China; 4 Key Laboratory of Modern Preparation of Traditional Chinese Medicine, Ministry of Education, Jiangxi University of Chinese Medicine, Nanchang 330004, China

**Keywords:** aquaporins, AQP7, health and disease

## Abstract

Aquaporins (AQPs) are highly conserved small transmembrane proteins that facilitate the transport of water and small solutes across cell membranes. Aquaglyceroporin 7 (AQP7), a significant member of the AQP family, is widely distributed throughout the body. For years, AQP7 was predominantly recognized for its role as a small-molecule transporter, facilitating the passage of small molecular substances. However, growing studies have revealed that AQP7 is also involved in the regulation of lipid synthesis, gluconeogenesis, and energy homeostasis, and it is intimately linked to a variety of diseases, such as obesity, type 2 diabetes mellitus, cardiovascular diseases, cancer, and inflammatory bowel disease. This article presents a comprehensive overview of the structure of AQP7, its regulatory mechanisms, its vital roles in both healthy and diseased states, and potential therapeutic advancements. We hope that these studies will serve as a valuable reference for the development of future treatments and diagnostic protocols targeting AQP7.

## 1. Introduction

Maintaining water homeostasis in the body is critical for all organisms, and one of the most prominent ways to transport water is to rely on the aquaporins (AQPs). AQPs are a family of small, integral membrane proteins in mammals. Since the first discovery of AQP1 by Nobel Laureate P. Agree [1,2], there has been an explosive growth in research on AQPs. AQPs are mainly responsible for the transportation of water and small uncharged molecules like glycerol, urea, CO_2_, NH_3_, and H_2_O_2_ [3,4]. A total of 13 AQPs have been identified in mammals, and they are found in various tissues. AQPs can be categorized into three primary groups based on their structure and permeability: orthodox aquaporins, aquaglyceroporins and superaquaporins.

Orthodox aquaporins are mainly responsible for transporting water, and the predominant types include AQP0, AQP1, AQP2, AQP4, AQP5, AQP6, and AQP8. Aquaglyceroporins facilitate the transportation of water and other small, uncharged molecules, like urea and glycerol. The primary aquaglyceroporins include AQP3, AQP7, AQP9, and AQP10. Superaquaporins are a special type of aquaporin, exhibiting very low homology compared to other AQPs. Some controversy remains over the substances transported by superaquaporins. This type of aquaporin can participate in modulating intracellular water, glycerol and hydrogen peroxide transport, the regulation of organelle volume, and the maintenance of vesicle homeostasis, including AQP11 and AQP12 [5,6,7].

AQP7, as a prominent member of the AQP family, stands among the most comprehensively researched aquaporins. It is mainly distributed in adipose tissue, the pancreas, muscles, the kidneys, skin, the intestines, and other tissues and organs. Although the current understanding of AQP7 is primarily confined to its function as a glycerol transporter, an increasing number of studies have revealed that AQP7 is involved in the regulation of lipid biosynthesis, gluconeogenesis, and energy homeostasis within the normal body [8,9,10]. Furthermore, it plays a significant role in various diseases, such as obesity, type 2 diabetes mellitus (T2DM), cardiovascular diseases, cancer, and inflammatory bowel disease. Thus, this article provides an overview of the latest advancements in the structure and distribution of AQP7 under normal physiological conditions, summarizes the regulatory mechanisms of AQP7 mediated by various substances for the first time, and emphasizes the crucial role of AQP7 in health and disease.

## 2. Structure and Tissue Distribution

**Figure 1:**
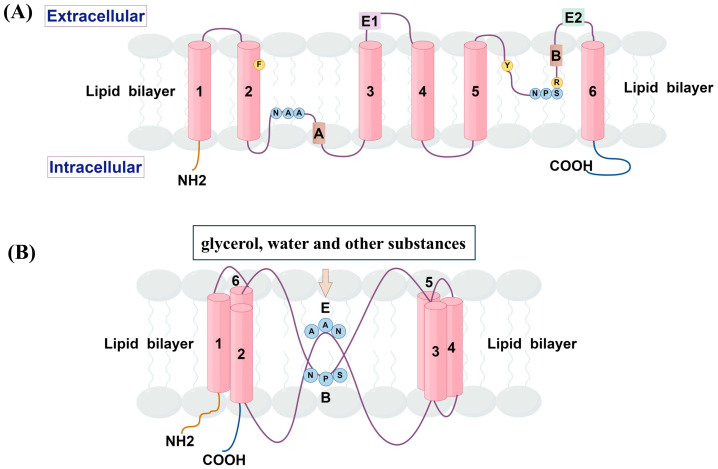
A secondary structure and topology of AQP7 molecule (by Figdraw (www.figdraw.com)). (**A**) AQP7 monomer has six membrane-spanning regions (1–6), extracellular helices E1 and E2, and membrane-embedded helices A and B with intracellular ammino and carboxy termini, as well as internal tandem repeats. (**B**) In the monomer, water transport and selectivity are facilitated by both the NAA and NPS motifs and the Ar^2^/R filter.

Despite the large variation in amino acid sequences, the structure of AQPs is highly conserved. AQP7 has the basic structure of the AQP family, which exists as a tetrameric fold, with each subunit consisting of six transmembrane helixes and two short helixes. The amino and carboxyl termini are located within the cell, forming a barrel-like structure, with each subunit contributing to a separate glycerol channel [11,12]. However, unlike most human AQPs, the asparagine–proline–alanine (NPA) motif and aromatic/arginine (Ar/R) sites related to the material selectivity have changed. Furthermore, the NPA motif was altered in all amino acids, except for retaining asparagine, which was replaced to form NAA (Asn94, Ala95 and Ala96) and NPS (Asn226, Pro227 and Ser228) motif, respectively. Meanwhile, the presence of the aromatic residue Y223 strengthens the Ar/R filter arising from the F74-R229 contact, leading to the formation of a more robust Ar^2^/R filter with enhanced cation–π interaction [13,14]. The structure is shown in Figure 1.

AQP7 was first discovered in the testes of 9-month-old Wistar rats in 1997 [15], and human AQP7 was identified within adipose tissue during the same year [16]. Subsequently, it was discovered in various tissues and organs of mammals, including distribution in brown adipose tissue, the pancreas, skeletal muscle, cardiac muscle, the kidneys, and the intestines. The specific distribution and localization of AQP7 in humans and rodents are shown in Table 1.

## 3. Role of AQP7 in Health

**Figure 2:**
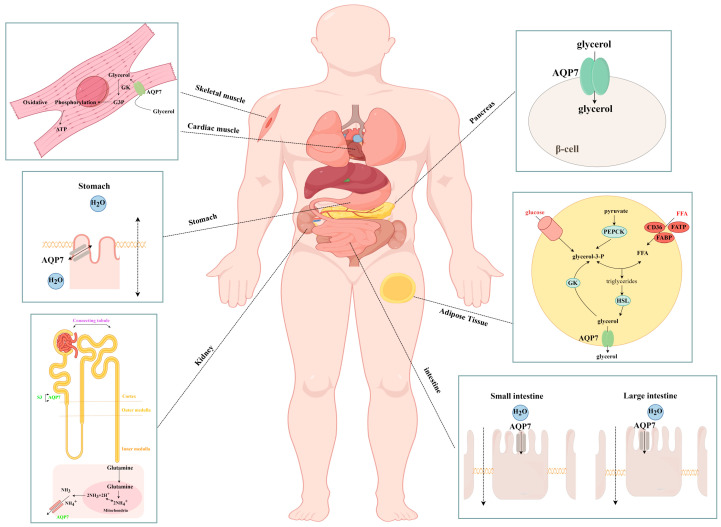
Transport of glycerol and other substances by AQP7 in different tissues (by Figdraw).

**Figure 3:**
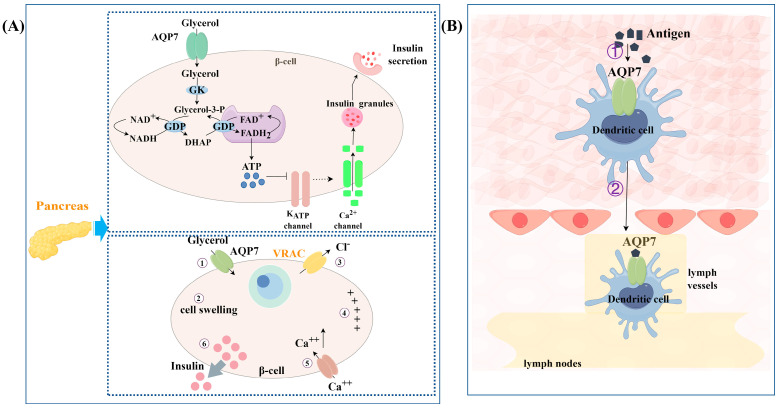
(**A**) Regulation of insulin release by AQP7 in β-cells; (**B**) involvement in immune responses by AQP7 in skin dendritic cells. ① Antigen uptake by AQP7; ② migration into lymphatic system. All figures created by Figdraw.

**Figure 4:**
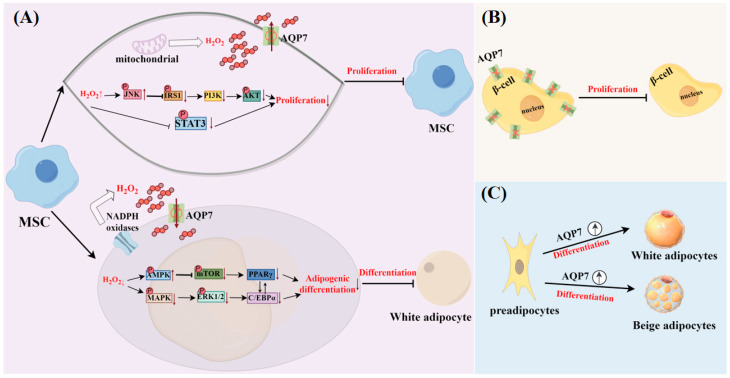
Facilitation of cell differentiation and proliferation by AQP7 (by Figdraw). (**A**) Bone marrow mesenchymal stem cells; (**B**) β cells; (**C**) adipocyte cells.

AQP7 exhibits a widespread distribution throughout various tissues and organs within the body. Besides its function in transporting glycerol and other substances, it also performs other roles, such as regulating insulin release, participating in immune responses, and facilitating cell proliferation and differentiation. The role of AQP7 in health is illustrated in Figure 2, Figure 3 and Figure 4.

### 3.1. Transport of Glycerol and Other Substances

The primary function of AQP7 is to transport glycerol, which has been observed to cross cellular membranes in diverse tissues, including adipose tissue [35], the pancreas [25], skeletal [36,37] and cardiac muscles [22], and the kidneys [27,38] (Figure 2). Glycerol is then catalyzed by a variety of enzymes, including glycerol kinase (GK) and glycerol-3-phosphate dehydrogenase-2, and subsequently participates in energy conversion and storage. This process significantly contributes to maintaining the organism’s energy balance and homeostasis [35,39,40,41]. Additionally, AQP7 has also been found to facilitate the transportation of water and other small molecules, particularly within the gastrointestinal system [29]. AQP7 may also have a role in the transportation of NH_3_ or NH^4+^ in the kidneys [42], although the specific mechanism of this transport is not yet fully understood. It can be hypothesized that this is possibly due to the presence of glutamine metabolism in the proximal renal tubules [43].

### 3.2. Regulation of Insulin Release

AQP7 is believed to have a regulatory effect on insulin release (Figure 3A). There is a certain relationship between this promoting effect on insulin release and the transport of glycerol. When β cells are subjected to an extracellular isotonic glycerol addition, glycerol enters the β cells through AQP7, causing themβ cells to swell. The increase in cell volume activates the volume-regulated anion channel (VRAC), consequently resulting in the depolarization of the plasma membrane and ultimately triggering the release of insulin [26]. Meanwhile, in the pancreases of AQP7^−/−^ mice, an elevation in glycerol content was observed with a proportional augmentation in GK activity, ultimately leading to a dramatic elevation of plasma insulin levels. It is speculated that the absence of AQP7 in the pancreas leads to the accumulation of glycerol aggregates in the pancreas. As GK activity increases, it drives the conversion of glycerol to glycerol-3-phosphate (G3P), which then enters the G3P shuttle pathway. In this pathway, G3P is catalyzed to generate ATP via the mitochondrial glycerol-3-phosphate dehydrogenase (GPD) enzyme, which is located within the inner membrane. The subsequent increase in the cytosolic ATP/ADP ratio results in the closure of ATP-sensitive potassium channels, leading to depolarization of the plasma membrane. This depolarization then activates voltage-gated calcium channels, resulting in a fast influx of calcium ions. The increase in calcium ions promotes the release of insulin [44]. Additionally, there is an enhanced absorption and utilization of glucose in AQP7^−/−^ mice, leading to an elevation in the ATP-to-ADP ratio. This enhances the synthesis and release of insulin in turn [25]. Recent studies have revealed that AQP7 forms a dimer of tetramers in human pancreatic β cells. This structure functions as intercellular adhesion proteins within the endocrine pancreas and promotes the rosette-like structure formation of β cells around blood capillaries, which is crucial for the regulation of insulin release [32].

### 3.3. Involvement in Immune Responses

AQP7 is also involved in immune responses (Figure 3B). This study found that the absorption of small molecules (fluorescein isothiocyanate and luciferase) and large molecules (ovalbumin and dextran) was reduced in AQP7-deficient skin dendritic cells (DCs). Meanwhile, a diminished capability for chemokine-dependent cell migration was displayed in AQP7-deficient DCs compared to wild-type DCs. Consistent with the in vitro findings, a decrease in the accumulation of antigen-retaining DCs was observed in the lymph nodes following the administration of antigen to the skin of AQP7^−/−^ mice. All of these findings suggest that AQP7 mainly has a role in capturing antigens and facilitating the migration of DCs. Furthermore, it is also responsible for presenting antigens and stimulating immune responses downstream. The mechanism by which AQP7 is involved in the immune response may be related to its mediation of water efflux, which controls cell volume during micropinocytosis [23].

### 3.4. Facilitation of Cell Differentiation and Proliferation

AQP7 facilitates the processes of cell differentiation and proliferation (Figure 4). It was shown that the level of AQP7 mRNA rose as the 3T3-L1 adipocytes differentiated [45]. The same phenomenon also occurs during the differentiation process of beige adipocytes. The level of AQP7 mRNA was increased as beige adipocytes differentiated, but the magnitude of the increase was significantly lower than that of white adipocytes undergoing the same differentiation process. This could be linked to the physiological role of beige adipocytes, particularly their requirement for oxidative metabolism to produce more heat [46]. AQP7 expression was also found to be associated with cell proliferation in both AQP7^−/−^ mice and RIN-m5F cells. A reduced β-cell mass was observed in AQP7^−/−^ mice, and a higher cell proliferation rate was presented when AQP7 was overexpressed in RIN-m5F cells [47]. AQP7 was also found to be closely related to the proliferation of bone marrow mesenchymal stem cells (BMSCs), as well as their differentiation into adipocytes, both of which are associated with the transport of H_2_O_2_ by AQP7 in BMSCs. Under normal proliferation conditions, AQP7 deficiency can result in elevated amounts of H_2_O_2_ within the cell, causing oxidative stress and blocking PI3K/AKT and STAT3 signaling pathways. This further impairs the proliferation of BMSC. It was also found that the adipogenic differentiation ability of AQP7-deficient BMSCs was significantly reduced. This may be due to AQP7 deficiency resulting in alterations in the AMPK and MAPK signaling pathways, as well as decreased expression of the lipogenic genes CCAAT/enhancer binding protein–alpha (C/EBP-α) and peroxisome proliferator-activated receptor γ (PPARγ) [48].

## 4. Regulation of AQP7 by Hormones and Non-Hormonal Substances

**Figure 5:**
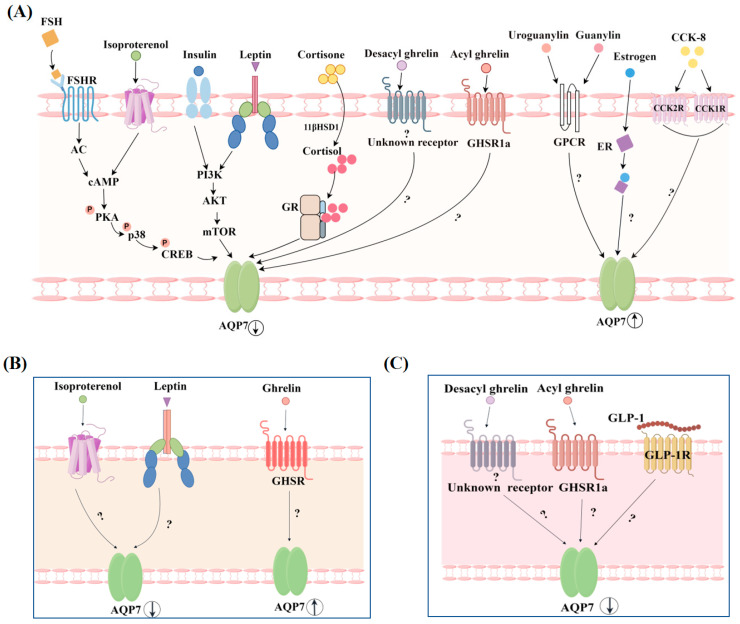
Regulation of AQP7 expression by hormones (by Figdraw). (**A**) White adipocyte; (**B**) brown adipocyte; (**C**) pancreatic β cells.

**Figure 6:**
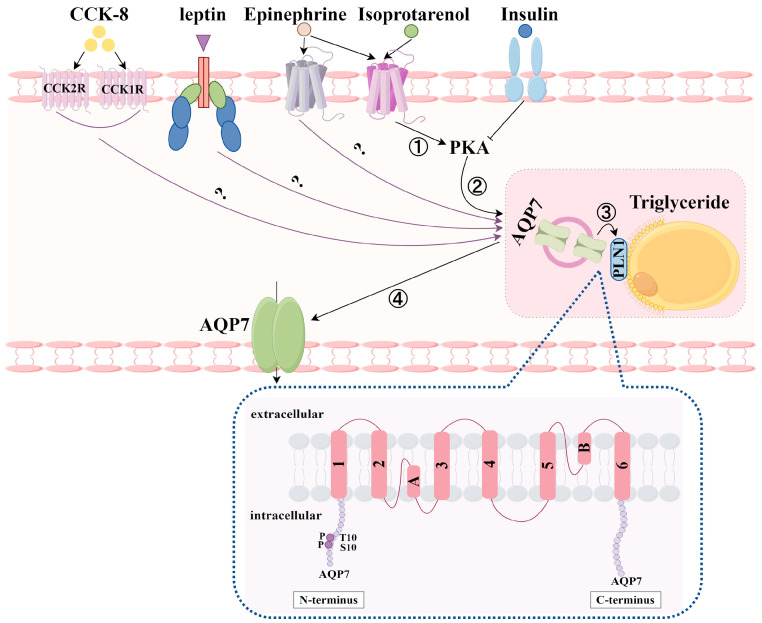
Regulation of AQP7 translocation by hormones (By Figdraw); ① Isoproterenol activates PKA; ② PKA phosphorylates the S10/T11 region of AQP7’s N-terminal; ③ the binding capacity between PLIN1 and AQP7 weakened by PKA; ④ AQP7 translocates to the plasma membrane.

**Figure 7:**
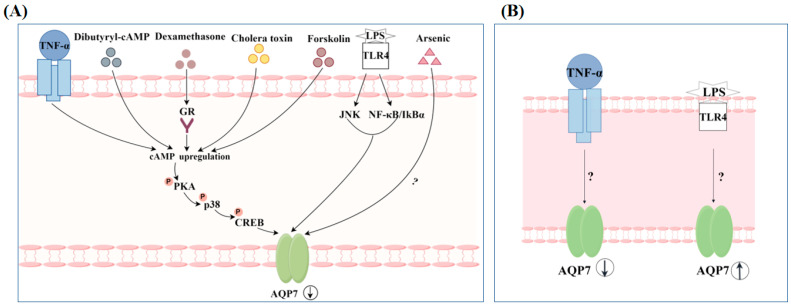
Regulation of AQP7 expression by non-hormonal substances (By Figdraw). (**A**) White adipocyte; (**B**) pancreatic β cells.

Like in other AQPs, the localization and expression level of AQP7 are also controlled by several variables, including hormones and non-hormonal substances. These substances play a pivotal role not only in maintaining the normal state of the organism but also in the development and progression of diseases, being closely linked to health and disease states. This could be the crucial reason why AQP7 assumes a significant role in both health and disease conditions. The specific regulatory processes are shown in Figure 5, Figure 6 and Figure 7.

### 4.1. Regulation of AQP7 by Hormones

Glycerol not only serves as the main chain of triglycerides (TGs), but also as the precursor of carbohydrates, lipids, and phospholipids. Furthermore, it participates in glycolysis in the form of G3P [49]. AQP7, as the primary transporter of glycerol, has been found to be regulated by certain hormones involved in glycolipid metabolism in both its expression level (Figure 5) and its translocation process (Figure 6).

#### 4.1.1. AQP7 Expression Levels Regulated by Hormones

- Insulin

Insulin serves as the principal controller of carbohydrate, lipid, and protein metabolism. The increased glucose concentration in the blood plasma stimulates β cells to secrete insulin after maintaining the balance of glucose in the body [50]. Prior research has demonstrated that the expression of AQP7 mRNA in adipose tissues of mice exhibited an increase during periods of fasting, while it was decreased during feeding. This change coincided with opposite plasma insulin levels in mice. It was also found that insulin suppressed AQP7 mRNA expression in 3T3-L1 cells in a manner that depended on the dosage [45]. Furthermore, when dietary obese C57BL/6 mice were administered with insulin for 4 weeks, the protein expression of AQP7 in their adipose tissue showed a downward trend [51]. These findings indicate a potentially significant correlation between insulin and the expression of AQP7. The potential cause of this phenomenon could be attributed to the presence of an insulin-response element (IRE, −443 to −437) within the promoter region of the mouse AQP7 gene. Three IREs (−121/−115, −629/−623, −542/−536) have also been discovered in the human AQP7 gene. However, it is surprising that the expression of AQP7 increased in human omental adipocytes following insulin treatment [52,53]. This difference in the degree of AQP7 expression may be related to species specificity. In further exploring the mechanism of the insulin regulation of AQP7, it was found that both the phosphatidylinositol 3-kinase (PI3K) inhibitor, wortmannin, and the mammalian target of rapamycin (mTOR) inhibitor, rapamycin, could inhibit the regulatory effect of insulin on AQP7 in human omental adipocytes. Hence, there is speculation that insulin can control the expression of AQP7 via stimulating the PI3K/Akt/mTOR signaling pathway [54].

- Leptin

Leptin is a hormone produced and secreted by adipose tissue. It plays a role in controlling the balance of energy and body weight in organisms by decreasing appetite and boosting energy expenditure [55]. Upon leptin treatment, a downregulation of AQP7 mRNA expression was observed in human omental adipocytes, which may be related to the decrease in lipid droplet levels within adipocytes caused by leptin [54]. To gain a deeper understanding of this regulatory relationship, researchers subsequently turned to leptin-deficient obese ob/ob mice for their studies, with the goal of elucidating the complex ways in which leptin regulates AQP7. AQP7 expression was also significantly upregulated in the subcutaneous white adipose tissue of ob/ob mice. Following a 4-week administration of leptin to ob/ob or wild-type mice, it was shown that the expression of AQP7 in the subcutaneous white adipose tissue of the mice decreased. This regulation may be related to the significant reduction in PPARγ transcription levels after long-term leptin administration [56]. Given that previous studies have demonstrated that there is a positive correlation between the expression of AQP7 and the transcription level of PPARγ, it follows that a decrease in the transcription level of PPARγ would subsequently lead to a reduction in AQP7 expression [53]. In addition, it has also been found that both the PI3K inhibitor, wortmannin, and the mTOR inhibitor, rapamycin, can inhibit the regulatory effect of leptin on AQP7 in human omental adipocytes. This leads to the speculation that the regulatory mechanism of leptin on AQP7 is similar to that of insulin, where leptin may also regulate AQP7 expression through the activation of the PI3K/Akt/mTOR signaling pathway [54]. Recently, there have also been studies investigating the modulation of AQP7 in brown adipocytes by leptin. When differentiated rat brown adipocytes were stimulated with leptin, AQP7 expression was dramatically downregulated. This phenomenon could be associated with the negative feedback regulatory mechanism via which leptin stimulates the sympathetic nervous system, consequently limiting the release of glycerol from brown adipocyte tissue [57].

- Cortisone

Under physiological conditions, glucocorticoids can promote lipolysis. However, excessive glucocorticoids can lead to obesity, which is associated with the overexpression of 11β-hydroxysteroid dehydrogenase 1 (Hsd1). This enzyme catalyzes the conversion of inactive cortisone to active cortisol, thereby causing obesity [58,59,60]. When cortisone was administered to 3T3-L1 cells, it caused a gradual decrease in the expression of AQP7 mRNA over time. This inhibitory effect may potentially be attributed to the involvement of Hsd1 [61,62].

- Cholecystokinin

Cholecystokinin (CCK), a peptide hormone, is released by I cells in the small intestinal mucosa. Its main role is to stimulate the pancreatic acini to secrete various digestive enzymes, and to promote gallbladder contraction for bile excretion. Recent research has found that CCK can promote white adipose tissue expandability and enhance the production and release of adiponectin, a hormone that improves insulin sensitivity. It has a vital function in maintaining the homeostasis of white adipose tissues (WAT) [63,64]. After treatments with the bioactive fragment of CCK-8 on Sprague–Dawley rats and preadipocytes, the AQP7 mRNA expression was observed to have increased. This regulatory effect was believed to rely on the activation of protein kinase B and PPARγ, as well as the integrity of insulin receptors in adipocytes [65].

- Ghrelin

Ghrelin, a hormone composed of peptides, is synthesized by X/A-like cells within the oxyntic glands of the gastric fundus mucosa, which has the effect of stimulating growth hormone secretion, stimulating caloric intake, increasing body weight, and promoting obesity. This hormone mainly circulates in the body in two forms: desacylated ghrelin (95% of the total ghrelin) and acylated ghrelin (5% of the total ghrelin) [66,67,68]. When both forms of ghrelin were exposed to differentiating visceral adipocytes, it was found that the AQP7 mRNA levels were suppressed by both acylated and desacylated ghrelin. This may also be related to ghrelin’s ability to reduce fat decomposition and promote the enlargement of adipocytes [69]. However, the opposite phenomenon was observed when ghrelin acted on rat brown adipocytes. Ghrelin upregulated the expression of AQP7 which may be related to promoting glycerol influx for maintaining the substrate for TG storage in thermogenic cells [57]. It is noteworthy that ghrelin not only regulates AQP7 in adipocytes, but also exerts a similar regulatory effect on AQP7 in pancreatic cells. Research revealed that the exposure of pancreatic the β-cell line RIN-m5F to acylated and desacylated ghrelin resulted in the decreased expression of AQP7. This finding indicates that the reduction in AQP7 caused by ghrelin may lead to the accumulation of intracellular glycerol, which could be further utilized for the biosynthesis of TG and the generation and release of insulin in β cells [70].

- Uroguanylin and guanylin

Uroguanylin and guanylin are secreted by intestinal epithelial cells in their prohormone forms. These hormones influence the hypothalamus, triggering a feeling of satiety [71,72]. When these hormones were incubated with differentiated human omental adipocytes, the expression of AQP7 was raised. This modulation may be linked to the promotion of lipolysis mediated by cAMP and cGMP signaling cascades in differentiated adipocytes [73].

- Sex hormones

Sex hormones have also been found to influence the expression of AQP7 in adipose tissue, and this effect exhibits tissue specificity. In both the visceral adipose tissue and the subcutaneous adipose tissue of ovariectomized mice, significant increases in fat depot mass and adipocyte size were observed. However, only a reduction in AQP7 expression and an elevation in GK expression were found in visceral adipose tissue. Administering estrogen as a supplement to mice whose ovaries had been removed selectively restored the expression of AQP7 and reduced GK expression in visceral adipose tissue [74]. This regulatory mechanism may be related to the presence of multiple estrogen response elements (EREs) in the AQP7 gene promoter. Therefore, it is hypothesized that estrogen may regulate AQP7 transcription by binding to EREs in the AQP7 gene promoter [75].

- Follicle-stimulating hormone

Follicle-stimulating hormone (FSH) is a type of glycoprotein hormone that originates from the pituitary gland. Its primary role is to regulate the growth of gonads and germ cells, as well as the synthesis of estradiol [76]. When FSH acts on mature 3T3-L1 adipocytes and human primary adipocytes, the levels of both AQP7 mRNA and protein expression decrease. This phenomenon is primarily attributed to the presence of the activator protein-1 response element (AP1-RE) in the AQP7 promoter. Previous studies showed that the expression of cAMP-responsive-element-binding protein (CREB) was increased when mature 3T3-L1 adipocytes were exposed to FSH. Hence, FSH then increased the interaction between CREB and the AP-1 site, while simultaneously diminishing the interaction between the transcriptional activation factor c-Jun and the AP-1 site. This process ultimately leads to a reduction in the transcription and expression of AQP7 [77].

- Isoproterenol

Isoproterenol has also been found to regulate the expression of AQP7. The expression of AQP7 mRNA shows dose-dependent inhibition after treatment with isoproterenol in 3T3-L1 adipocytes. This reduction may be attributed to the elevation of cAMP levels, which occurs due to the activation of GS-protein and adenylate cyclase by isoproterenol. High levels of cAMP subsequently further activate lipolysis, thereby inhibiting the expression of AQP7 and limiting the release of glycerol from adipocytes, in order to maintain the balance of glucose and lipids. However, this regulatory effect seems to require further investigation [54,61]. The impact of isoprenaline on AQP7 regulation was also noted in brown adipocytes. Following the administration of isoproterenol to differentiated rat brown adipocytes, it was found that isoproterenol significantly downregulated the AQP7 mRNA level in these cells. This regulatory mechanism is analogous to the mechanism by which leptin regulates AQP7 in brown adipocytes, which further limited glycerol release from brown adipocytes when sympathetic nerves were activated by isoprenaline [57].

- Glucagon-like peptide-1

Glucagon-like peptide-1 (GLP-1) stimulates the secretion of insulin from pancreatic β cells and inhibits the secretion of glucagon from pancreatic α cells in a manner that depends on the concentration of glucose, thereby lowering blood glucose levels. Recent studies have shown that GLP-1 also showed a tendency to downregulate AQP7 expression when stimulating RIN-m5F β cells. It is speculated that the GLP-1-induced reduction in AQP7 expression may lead to intracellular glycerol accumulation, and the accumulated glycerol may then be further utilized for TG biosynthesis, as well as for the production and release of insulin in β cells [72].

#### 4.1.2. Translocation of AQP7 by Hormones

In addition to regulating the abundance of AQP7 mRNA and protein expression, hormones can also modulate the AQP7 translocation process (Figure 6). After treatments with isoproterenol [54], epinephrine [45], leptin [56], and CCK-8 [65] on adipocytes, AQP7 was found to be transferred from the intracellular region to the plasma membrane. When 3T3-L1 adipocytes are stimulated with insulin, AQP7 more noticeably surrounds the lipid droplets (LDs) [54]. Interestingly, the results of this translocation are not entirely consistent in other studies. Some researchers have found that in WAT from mice exposed to insulin and norepinephrine, AQP7 stimulated by insulin is localized to the adipocyte cortex, while AQP7 stimulated by norepinephrine appears in the intracellular region surrounding the LDs [18]. This variation could be attributed to differences in cell types and experimental manipulation methods. This translocation phenomenon has also been found in human adipocytes. After the stimulation of human primary adipocytes with isoproterenol, it was found that AQP7 was also able to move from the LDs to the plasma membrane. The translocation mechanism may be due to the fact that isoproterenol activates protein kinase A (PKA), which results in the phosphorylation of the S10/T11 region of AQP7′s N-terminal. This phosphorylation weakens the binding capacity between PLIN1 and AQP7, facilitating the translocation of AQP7 to the plasma membrane [78,79,80,81]. The phenomenon of the insulin-induced translocation of AQP7 may also be related to the regulation of PKA. AQP7 obviously also surrounds the LDs after insulin stimulates human primary adipocytes, and the number of AQP7-PLIN1 complexes formed after insulin stimulation is greater than the number of AQP7-PLIN1 complexes formed after isoprenaline stimulation. This phenomenon may be related to insulin’s ability to inhibit PKA activity [80,81].

### 4.2. Regulation of AQP7 by Non-Hormonal Substances

Peroxisome proliferator-activated receptors (PPARs), as ligand-activated transcription factors, belong to the nuclear hormone receptor superfamily. They play a role in regulating the balance of energy, glucose, and lipid metabolism to maintain homeostasis. PPARs include three main subtypes: PPARα, PPARγ, and PPARβ/δ [82]. PPARα is considered to be closely related to maintaining lipid metabolic homeostasis. In PPARα-deficient mice, the elevation of AQP7 transcription levels in adipose tissue caused by fasting is blocked [83]. PPARγ is crucial for adipocyte differentiation. When the PPARγ agonist pioglitazone was used to treat mice or 3T3-L1 adipocytes, it was found that AQP7 mRNA expression was increased. Simultaneously, AQP7 mRNA expression was significantly reduced in PPARγ-deficient mice. This regulatory process may be related to the presence of the peroxisome proliferator response element (PPRE) on the promoter of the mouse AQP7 gene. After PPARγ is activated, it interacts with the retinoic acid X receptor to form a heterodimer, which then directly binds to the PPRE on the AQP7 gene promoter, thereby activating AQP7 expression. PPRE is located at −93 to −77 on the mouse AQP7 gene promoter [84], and PPRE has also been found at −46 to −62 on the human AQP7 gene promoter [53].

In addition, some substances have been found to regulate the concentrations of AQP7 mRNA or protein expression in adipocytes as well. After exposing 3T3-L1 cells to tumor necrosis factor (TNF-α), the Gs protein activator cholera toxin, the adenylate cyclase stimulant forskolin, dexamethasone, and the cAMP analog dibutyryl-cAMP, it was observed that AQP7 mRNA expression was inhibited, suggesting that intracellularly accumulated cAMP may be a potential key mediator for these substances to regulate AQP7 expression [61]. Arsenic has also been found to affect AQP7 expression levels. After administering arsenic to C57BL/6 male mice, a decreasing trend in AQP7 was found in both visceral white adipose tissue (epididymal and retroperitoneal) and subcutaneous white adipose tissue, with the perirenal white adipose tissue the most affected [85]. When 3T3-L1 cells are incubated with LPS, AQP7 expression shows a decreasing trend. Furthermore, the regulatory mechanism may be influenced by activating the TLR4-induced JNK/NF-κB signaling pathway [86]. Although TNF-α and LPS were also used to treat RIN-m5F β cells, the trend in regulating AQP7 expression in these cells differs from that in adipocytes. TNF-α caused a decreasing trend in AQP7 mRNA levels in RIN-m5F β cells, whereas LPS showed an upregulating trend in AQP7 mRNA levels in RIN-m5F β cells. The specific mechanisms of how these substances regulate AQP7 still remain to be further studied [47] (Figure 7).

## 5. Involvement of AQP7 in Multiple Diseases

### 5.1. Obesity

As early as in AQP7 knockout (AQP7-KO) mice, it has been found that the knockdown of AQP7 promotes a decrease in glycerol permeability in adipocytes, resulting in a rise in glycerol accumulation in adipocytes. The accumulated glycerol induced the rise in GK activity, further leading to the increase in TG synthesis in adipose tissue. This gave rise to adipocyte hypertrophy and a marked gain in body weight. This increase in adiposity was not related to adipogenesis or lipolysis, as lipoprotein lipase activity (a key lipolysis regulator) and PPARγ or C/EBPα expression (critical adipogenesis factors) remain unchanged in the absence of AQP7. However, this phenomenon appeared only in the adipose tissues of adult mice (after 10 weeks age) [87,88]. Intracellular TG accumulation in adipocytes was also observed in AQP7-knockdown 3T3-L1 adipocytes, and it was proposed that the decrease in AQP7 expression might be linked to the intracellular accumulation of lipids, contributing to an increase in membrane surface tension [17]. The above results indicate that a reduction in AQP7 expression or function in adipocytes may be associated with increased obesity. Interestingly, the findings from studies on two other AQP7 KO mouse lines were slightly different, showing no change in adipocyte size or body weight [25]. Additionally, plasma glycerol levels remained similar in both AQP7-KO and wild-type mice [20,25]. The potential reasons for these discrepancies may include differences in the genetic backgrounds of the mice, as well as variations in tissue sample collection and analysis methods. Some studies have also found AQP7-promoter variants in obesity. In a study of Caucasian childhood obesity, the single-nucleotide polymorphisms (SNPs) A2710G, C2782A, C2910A, G3069C, C3117T, and T2816A were identified in the promoter region of the AQP7 gene. Among them, the most noteworthy is the T2816A SNP. Studies have indicated that the level of AQP7 mRNA expression in prepubertal obese children carrying this mutant gene is lower than that of other prepubertal children with obesity, although their fasting insulin concentration is normal. Additionally, their fasting serum glycerol concentration is higher than that of lean children and those with obesity who are the same age. This leads to the speculation that this mutation possibly contributes to the obese phenotype, since the mutation region is located between the binding sites of transcription factors CRE-BP and C/EBPα, and it is speculated that the mutation site might serve as a regulatory site for transcription factor SP1. However, whether this mutation is connected to the capacity of the cAMP response element to stimulate AQP7 mRNA expression remains to be further studied [89]. This result further indicates that there is a close relationship between AQP7 and obesity.

To further explore the relationship between AQP7 and obesity, a study was conducted focusing on the association between AQP7 and various tissues, ages, and genders in obese patients and rodents. Similar to the phenomenon noted in the white adipose tissue of AQP7 KO mice, the amounts of AQP7 mRNA and protein were diminishing in the abdominal subcutaneous white adipose tissues of individuals with different levels of obesity [90,91]. However, the degree of change in AQP7 mRNA levels in visceral tissues of obese patients is not entirely consistent. Some obese patients exhibited a decreasing trend in the concentrations of AQP7 mRNA or protein in visceral white adipose tissue [90], while others showed an increasing trend in AQP7 mRNA levels [92,93]. This may be attributed to significant differences at the genetic level in omental adipose cells among humans. More interestingly, the AQP7 mRNA level in the visceral white adipose tissue of obese patients is generally significantly higher compared to that in subcutaneous white adipose tissue. This could be related to the greater sensitivity of visceral adipose tissue to catecholamine-induced lipolysis and its higher basal lipolysis rate compared to subcutaneous adipose tissue [93,94,95]. The level of AQP7 expression in brown adipose tissue is also altered in obesity. When in a state of obesity, brown adipocyte tissue differentiates into white-like unilocular cells, which is referred to as brown adipose tissue whitening. These brown adipocytes often exhibit hypertrophy, fatty degeneration, and increased expression of lipogenic enzymes, resulting in the reduced functional activity of brown adipocytes [96]. This phenomenon may be related to the upregulation of AQP7 mRNA in brown adipocytes. The upregulated AQP7 promotes the influx of glycerol from the capillary endothelial cells of brown adipose tissue into the brown adipocytes, further leading to hypertrophy of the brown adipocytes, and then to the whitening of brown tissues [76]. In addition to the extensive research on AQP7 in the adipose tissues of obese humans and rodents, AQP7 expression in skeletal muscle has gradually gained attention in studies on obesity. It is puzzling to note that the expression level of AQP7 in the muscle of ob/ob mice increased, differing from the expression trend observed in white adipose tissue. This difference may have a strong correlation with the absence of leptin in this type of mouse, because previous studies confirmed that leptin can reduce the expression of AQP7 through the PI3K/AKT pathway [54]. These results show that the degree of AQP7 expression in different tissues is not consistent even in the same disease. Whether this regulatory process depends on the release of different hormones in the body, or whether it is regulated by other mechanisms remains to be further studied [97].

The expression by adipocyte AQP7 of obesity at different ages also appears to be altered to different degrees, and even the AQP7 isoforms presented in adipose tissue vary among obesity patients of different ages. Upon detecting the AQP7 mRNA level in the abdominal subcutaneous adipose tissues of obese children in different age groups, it was found that the AQP7 mRNA increased in the younger obese prepubertal children. Conversely, there was a drop in AQP7 mRNA levels in the obese adolescents, which was accompanied by an increase in insulin resistance. More interestingly, multiple AQP7 isoforms were exhibited in the adipocytes of children and adolescents with obesity. These were the 34 kDa, 41 kDa, and 37 kDa isoforms of AQP7. Notably, the expression of the 41 kDa AQP7 isoform was only significantly increased in the adipose tissues of lean adolescents and younger obese prepubertal children, indicating that the AQP7 41 kDa isoform may serve as a protective mechanism [98]. The existence of the AQP7 34 kDa and 37 kDa isoforms has also been confirmed in the subcutaneous, abdominal, and visceral adipose tissues of adults with morbid obesity. The expression levels of these isoforms are markedly diminished, and the absence of 41 kDa isoforms confirms the close relationship between this subtype and age [90]. As a result, the regulatory mechanisms that govern the differential expression of AQP7 in adipose tissue in obesity necessitate further exploration.

There also appears to be a strong relationship between gender and AQP7 expression in obesity. Female and male C57BL/6JRj mice were given a high-fat diet (HFD) for a period of 12 or 24 weeks, respectively. In comparison to the male control group, the body weights of the male mice showed a significant increase, and adipocytes were significantly enlarged, regardless of whether they were fed a HFD for 12 weeks or 24 weeks. Compared to the female control group, while the body weights of female mice treated with HFD also increased significantly, only those female mice that were administered a 24-week HFD exhibited a significant enlargement of adipocytes. Additionally, it was observed that only the female mice exhibited an elevation in AQP7 protein expression in their adipose tissues. This suggests that the lower degree of obesity caused by short-term HFD in female mice compared to male mice may be linked to the elevated expression of AQP7 in the adipocytes of female mice [99]. A similar phenomenon was also observed in the adipose tissues of obese women. When comparing lean persons to obese women, it was observed that the AQP7 mRNA levels in the subcutaneous adipose tissue only slightly decreased in obese women, but significantly decreased in extremely obese women (BMI > 40 kg/m^2^) [100]. The AQP7 protein expression was also decreased in the subcutaneous and visceral adipose tissues of obese post-menopausal women [75]. Although studies have shown that there was a correlation between obesity and SNPs of the AQP7 gene only in female obesity [101], the levels of other substances related to the regulation of AQP7 expression were also changed in female obesity, such as an increasing trend in the expression levels of MCP1, TNF-α, and FSH [75] and a decreasing trend in PPARγ expression [100]. Therefore, further studies are required to determine whether genetic factors or other substances play a dominant role in the changes in AQP7 expression in different sexes and disease states.

In addition, there is a definite relationship between AQP7 gene promoter methylation and obesity. It was found that the promoter region of AQP7 exhibited hypomethylation at three CpG sites in diet-induced-obesity rat models. Furthermore, a negative correlation between the epididymal fat mass and the AQP7_CpG4 methylation was observed. The percentage of AQP7_CpG4 methylation also exhibited an inverse relationship with insulin plasma levels and leptin mRNA levels [102]. Nevertheless, an opposing outcome was noted in a separate investigation. It was also found that the AQP7 gene promoter contains CpG island diet-induced-obesity models in rats, but no change in AQP7 gene promoter methylation due to a high-fat, high-sucrose diet [103]. Hence, the precise regulatory mechanism linking obesity and AQP7 gene promoter methylation remains to be further elucidated.

### 5.2. T2DM and Its Complications

Some studies have also found AQP7 promoter variants in T2DM. The genotyping of four SNPs in AQP7 (rs2989924, rs3758269, rs3758268, and rs3758267) was observed in the subcutaneous adipose tissues of obese patients with T2DM. The AQP7 rs2989924 (A-953G) was suggested to be strongly associated with T2DM. This may be due to the fact that the A-953G variant negatively regulates AQP7 expression by reducing CCAAT/enhancer binding protein (C/EBP) β-DNA binding, and in turn impairs the biological function of AQP7-promoter transcriptional activity. However, this association was only observed in females. The cause of this gender-specific presence is uncertain, as it is unknown whether it is a result of the interaction between the AQP7 gene and gender-related genes or the effects of sex hormones [101]. The AQP7 SNPs rs2989924 and rs3758269 have been shown to have a significant correlation with T2DM in the Han Chinese population [104]. Nevertheless, contrasting findings have been reported in other studies, with some indicating that the AQP7 SNP rs3758269 is not associated with T2DM in the Chinese Han population. The differences between these research results may be related to the age of the sample selection, as the role of the AQP7 SNP rs3758269 in T2DM may become increasingly apparent with age [105]. In conclusion, all of these investigations indicate that AQP7 probably has a significant effect on T2DM.

To better explore the relationship between AQP7 and T2DM, researchers have also investigated the correlations among tissue, age, and AQP7 expression in both T2DM patients and rodents. Interestingly, similarly to the changing trend in AQP7 expression in subcutaneous adipose tissue in obesity, a significant decrease in the expression levels of multiple AQP7 isoforms such as the 37 kDa and 34 kDa isoforms of AQP7 was observed in the adipose tissues of T2DM patients with morbid obesity. These downregulated AQP7 levels may promote adipocyte enlargement and reduced basal lipolysis, which then possibly further leads to reduced cholesterol levels in plasma membrane caveolae [90], since reductions in cholesterol in the plasma membrane can induce the internalization of caveolins (the main structural protein of caveolae) [106], and this internalization subsequently impairs insulin signaling [107], which may be one of the reasons why obesity leads to T2DM. Additionally, the surprising finding of a significant negative correlation between the AQP7 34 kDa isoform and insulin, as well as HOMA-IR, indicates that the presence of the AQP7 34 kDa isoform in adipose tissue may have a major influence on insulin sensitivity. Moreover, a decrease in this isoform could potentially serve as an early marker for insulin resistance [90]. There is also the phenomenon of altered AQP7 levels in the visceral adipose tissues of obese patients with T2DM, but the obtained results are inconsistent. Some studies have found that the AQP7 mRNA levels in the visceral adipose tissues of obese patients with T2DM show a decreasing trend [94,108]. Others have observed an increasing trend in AQP7 mRNA levels in visceral adipocytes [92,93]. This difference in trends may be mainly attributed to the differences in the sensitivity of visceral adipose tissue to catecholamine-induced lipolysis [93], but it may also be related to different stages of T2DM. Several studies have found that the level of AQP7 expression in adipose tissue varies at different stages of T2DM. An increase in the expression of AQP7 mRNA and protein was reported in the perirenal adipose tissues of OLETF diabetic rats during the early stage of diabetes. This may be due to increased weight, which prompts the transportation of glycerol from adipose tissue into the liver for blood glucose synthesis, further elevating blood glucose levels. However, in the later stage of T2DM, the expressions of AQP7 mRNA and protein both declined. It is speculated that AQP7 may experience decompensation in the later stage of T2DM, leading to a reduction in the excretion of glycerol from adipocytes, which further aggravates obesity and exacerbates diabetic conditions [109].

Given skeletal muscle’s crucial role in maintaining glucose balance throughout the body due to its insulin sensitivity, AQP7 has also been implicated in the pathogenesis of muscle metabolic dysregulation in T2DM. The AQP7 mRNA expression was significantly elevated in the skeletal muscle of male db/db mice [110]. A similar phenomenon was found in the skeletal muscle of patients with T2DM. The level of AQP7 protein expression in the skeletal muscle of obese males with T2DM showed a notable increase compared to lean and healthy males. This rise may be linked to the inverse correlation between myocellular lipid accumulation and the sensitivity of the muscles to insulin. When AQP7 expression increases, glycerol intake also increases, which serves as a substrate for TG synthesis. This leads to an increase in TG content in the muscle and further reduces muscle sensitivity to insulin. However, whether the upregulation of AQP7 in muscle promotes the pathophysiological development of obese T2DM or is merely a response to the reduced insulin-mediated lipid oxidation inhibition in insulin resistance remains to be further studied [111].

Moreover, AQP7 expression varied significantly among T2DM rats of different ages. In the epididymal white adipose tissues of 22-week-old diabetic db/db mice, a declining trend in AQP7 mRNA expression was observed [112]. However, Kuriyama found the opposite result in their study using the same animal model, in which an increase in AQP7 mRNA levels was observed in the epididymal white adipose tissue of 9-week-old diabetic db/db mice [113]. This contradictory conclusion may be related to the differences in age in the db/db mice used by the two research teams. Prior research has demonstrated that the degree of insulin resistance decreases with age, and TNF-α may be increased with obesity duration. These factors may be the main reasons for the different levels of AQP7 expression in the WAT of db/db mice at different weeks of age [112].

AQP7 has additionally been implicated in the modulation of diabetic complications. Diabetes-impaired wound healing remains a critical therapeutic challenge. Impaired wound healing often leads to infections, chronic inflammation, sepsis, wound dehiscence, and even death [114]. In the diabetic model of STZ-induced rats, it was found that the expression level of AQP7 in wounds is decreased compared to normal controls, but the specific regulatory mechanism remains to be further investigated [115].

### 5.3. Cardiac Diseases

It has been found that the cardiac structure and function are normal under basal conditions in AQP7-KO mice. However, AQP7-deficient hearts exhibited severe left ventricular hypertrophy after being challenged with isoproterenol, and showed a higher mortality rate after transverse aortic constriction operation [22]. Additionally, studies on myocardial infarction in AQP7-KO mice revealed an increased area of myocardial infarction in their hearts, which may be related to glycerol transport. This is because glycerol is considered a substrate for energy production in cardiomyocytes under hypoxic conditions [116]. The expression of AQP7 mRNA is increased in hearts suffering from dilated cardiomyopathy. This rise in expression may be related to disorders of myocardial osmotic balance [117]. In vitro experiments also showed a notable increase in the level of AQP7 mRNA in a time-dependent manner after exposing H9c2 cells to hypertonic solution, but the degree of apoptosis may be mainly influenced by other aquaporins [118]. These findings suggest that the regulatory role of AQP7 in cardiac disease is likely to be primarily related to the regulation of the transport of glycerol or other substances.

### 5.4. Cancer

AQP7 is also thought to play a role in the occurrence and development of cancer. Breast cancer is the predominant form of cancer among women globally, accounting for around 25% of all cancer cases. Through the analysis of gene expression profiles in breast cancer among Indian women, it has been found to be related to changes in genes involved in lipid metabolism, particularly with AQP7 showing a downregulated state [119]. It is noteworthy that prior to the clinical manifestation of cancer, genes involved in lipid metabolism and adipogenesis are upregulated in susceptible breast tissues in women, with a particularly significant increase observed in AQP7 mRNA levels in these tissues [120]. Renal cell carcinoma (RCC), originating in the renal epithelium, comprises more than 90% of all renal cancers. Clear cell renal cell cancer (ccRCC) is the predominant form of renal cancer. The expression of AQP7 was markedly reduced in ccRCC patients [121]. Therefore, further research is needed to elucidate the precise regulatory mechanisms of AQP7 involvement in cancer occurrence and development.

### 5.5. Inflammatory Bowel Disease

Inflammatory bowel disease (IBD) is a persistent, recurrent disorder of the digestive system, commonly occurring in young adults and adolescents. IBD is distinguished by persistent stimulation of the immune system, accompanied by gastrointestinal inflammation and subsequent permeation of fluids, solutes, and lipids across the bowel mucosa. There are two clinical types: Crohn’s disease (CD) and ulcerative colitis (UC). It was found that AQP7 expression was significantly decreased in the DSS-induced mouse model of colitis and in patients with UC or CD. This reduction in AQP7 expression may serve as a negative feedback mechanism to counteract excessive water loss during inflammation in IBD. Meanwhile, the significant reduction in body weight of IBD patients may be related to glycerol transport through AQP7 or other AQPs [24,34].

## 6. AQP7 as Therapeutic Target

### 6.1. Treatment of Obesity

AQP7 plays a central role in anti-obesity. Following an 8-week period of administering apple polyphenols (APs) together with a HFD, AQP7 mRNA expression was significantly enhanced in rat adipose tissues. In addition, the AP supplementation not only promoted AQP7 expression, but also reversed the hypomethylation status of the three CpG sites in the promoter of the AQP7 gene induced by the high-fat and high-sucrose diet, further emphasizing the significance of AQP7 in resisting diet-induced obesity [102]. Resveratrol, another anti-obesity compound, achieves its fat-reducing effects primarily by promoting lipolysis, fatty acid oxidation, and thermogenesis. During this process, the expression of AQP7 is significantly increased, suggesting that resveratrol may accelerate fat loss through AQP7-mediated glycerol release [122]. Similarly, the positive effects of ginsenoside Rb1 (Rb1) on lipid metabolism were also notable in the regulation of AQP7. Studies have shown that Rb1 can reduce body weight, fat mass, and the adipocyte size and concentration of free fatty acids (FFAs) in the serum of mice with obesity induced by HFD. Meanwhile, Rb1 has been found to reduce lipid droplet accumulation and stimulate TG output in differentiated T3-L1 adipocytes. This regulatory process involves the upregulation of Rb1, which subsequently upregulates PPARγ and AQP7 protein levels [123]. Chitin is a plentiful mucopolysaccharide that occurs in the shells of crustaceans, the cuticles of insects, and the cell walls of certain fungi and bacteria. Its carboxymethylated derivative (CM-chitin) similarly demonstrated the potential to manage obesity by enhancing AQP7 expression. It was found that CM-chitin inhibits adipogenesis and promotes lipolysis by regulating the AMPK pathway and increasing the expression of AQP7 [124]. The nonsteroidal anti-inflammatory drug feprazone (4-prenyl-1,2-diphenyl-3,5-pyrazolidinedione) has demonstrated anti-obesity effects in clinical applications, with its mechanism potentially related to inhibiting adipocyte differentiation and promoting lipid metabolism. This process is accompanied by a significant increase in the expression levels of ATGL and AQP7, emphasizing the importance of AQP7 in facilitating lipid metabolism [125]. Raspberry ketone, an aromatic compound isolated from red raspberry (Rubus idaeus), has shown remarkable effects in alleviating diet-induced obesity. Its effects have also been hypothesized to be strongly correlated with increased AQP7 expression, further evidencing the central role of AQP7 in anti-obesity research [126].

Other methods, such as massage and sleeve gastrectomy, have also been found to have some effect on AQP7 in obesity tissues as a way to reduce fat accumulation. Abdominal massage has been found to significantly enhance the expression of AQP7 in the white adipose tissues of obese mice, subsequently reducing their fat mass, body weight, blood glucose, and lipid levels. It was hypothesized that abdominal massage could potentially improve the breakdown of fatty acids, regulate lipid metabolism, and maintain glucose balance by activating the PPARγ signaling pathway within the inguinal WAT. This might potentially provide therapeutic benefits in treating obesity [127]. In addition, sleeve gastrectomy, a surgical procedure, has shown positive effects on AQP7 in obesity treatment. By removing the fundus and larger curvature section of the stomach, the surgery not only effectively reduces body weight, but also exerts a sustained weight loss effect in humans and in models of obesity caused by genetics or diet. More importantly, studies have shown that this surgery can also reduce pancreatic β-cell apoptosis and improve insulin release, which may be associated with the increase in AQP7 expression in the pancreas. When AQP7 is upregulated, it can enhance the absorption of glycerol, leading to the increased synthesis and secretion of insulin [81].

### 6.2. Treatment of T2DM and Its Complications

AQP7 also plays a pivotal role in the treatment of T2DM and its complications. Metformin, the first-line drug for treating type 2 diabetes, has seen its latest anti-diabetic effects research focused on its positive impact on AQP7 expression in the pancreas. After administering metformin to STZ-induced diabetic rats and to pancreatic β cells (INS-1 cells) that had been harmed by hyperglycemia and hyperlipidemia, it was found that metformin can reverse the downregulation of AQP7 expression in islet cells caused by diabetes, and then promote an increase in glycerol entry into the pancreas and subsequent insulin secretion. The effect of metformin on regulating AQP7 is related to suppressing the p38 and JNK pathways [128].

Prior exposure to intense pulsed light (IPL) radiation enhanced the healing of wounds in diabetic rats, and it has also demonstrated positive effects mediated by AQP7 in the skin. After IPL pretreatment, it was shown that the expression of AQP7 in the wounded skin of diabetic rats increased to a level similar to that in normal rats. The wound-healing-promoting effect may be due to the fact that AQP7 participates in antigen uptake and subsequent DC migration. Additionally, AQP7 is responsible for the presentation of antigens and the facilitation of immune responses downstream. However, the specific regulatory mechanism remains to be further investigated [115].

### 6.3. Treatment of Cardiac Diseases

AQP7 also plays a significant role in the treatment of heart diseases. Coronary artery bypass grafting (CABG) is a very effective operation that is known for its ability to save the ischemic myocardium. However, since CABG utilizes extracorporeal circulation (CPB), it makes the myocardium prone to water imbalance and consequent edema. Clinical studies have found that diazoxide administration can alleviate edema following CABG. This mechanism may be related to the reduction in the relative expression of AQP7 during the surgery, which regulates water molecule transport in cardiomyocytes, thereby reducing edema [129].

Adriamycin (Dox) is a potent anticancer drug that is effective against several types of cancer. However, its clinical application is limited due to its adverse side effects, including severe cardiotoxicity. Cardiotoxicity can cause gradual and irreversible heart failure, which can lead to the development of interstitial myocardial edema. The Xinshuitong Capsule can alleviate the impairment of cardiac function, cardiac remodeling, and myocardial edema induced by Dox. The core mechanism of this protective effect is related to the inhibition of AQP expression in the myocardium, with a particular emphasis on significantly downregulating the level of AQP7 [130].

## 7. Conclusions and Future Perspectives

This article systematically reviews the structural and distributional characteristics of AQP7 under normal physiological conditions, and comprehensively summarizes its regulatory mechanism for the first time, based on the latest research results. Additionally, the article emphasizes the crucial role of AQP7 in maintaining health and disease states, providing valuable reference and guidance for future in-depth research on AQP7. Nevertheless, several key issues remain unanswered.

Although the function of AQP7 as a small-molecule transporter has been clarified and its structure is well known, the intricate regulatory mechanisms governing its transport process and the key regulatory factors involved still need to be fully elucidated. Thus, a profound exploration of the glycerol transport mechanism mediated by AQP7 is imperative for a deeper understanding of its role in health and disease.

Furthermore, AQP7 has been confirmed to play a pivotal role in regulating the pathogenesis of multiple diseases through the establishment of various AQP7-deficient animal models and cellular systems. However, the specific molecular mechanisms by which AQP7 participates in these disease-regulatory processes are still poorly understood. Future studies could aim to combine the regulation of AQP7 with hormonal signaling pathways that influence disease for a more comprehensive analysis. It is noteworthy that some studies have observed differences or even contradictions in the regulatory effects of AQP7 in the same disease, due to the selection of different stages of disease development as research nodes. In view of this, it is crucial to design and conduct larger-scale, scientifically rigorous, randomized, double-blind, and controlled preclinical and clinical trials to investigate the role of AQP7 in various diseases and its potential therapeutic value.

From a therapeutic perspective, AQP7 is considered to be a promising therapeutic target for a variety of diseases. Therefore, the development of appropriate in vitro models for screening and validating the function of AQP7 will significantly advance the translational research efforts in this field.

## Figures and Tables

**Table 1:** Localization of AQP7 in the tissues of humans and rodents.

Species	Tissue Localization	Subcellular Localization
Mice	visceral white adipose tissue [17,18], peritoneal cavity [19], interscapular brown adipose tissue [20]	adipocytes, stromal vascular fractions [17], endothelial cells [18], macrophage cells [19]
	kidneys [20]	segment 3 of murine renal proximal tubules [20]
	muscle [20]	capillary network of skeletal muscle, capillaries of cardiac muscle [20], the myofiber surface of type 1 and type 2 fibers [21], cardiomyocytes [22]
	spermatocytes [20]	the surroundings of mouse spermatids in tails, testicular and epipidymal spermatozoa tails [20]
	skin [23]	epidermal and dermal dendritic cells [23]
	colon [24]	basolateral aspect of the colonic epithelium [24]
	pancreas [25]	Β cells [25]
Rat	pancreas [26]	β cells [26]
	kidneys [22,27]	segment 3 proximal tubules [22,27]
	small intestinal [28,29]	superficial epithelial cells [28], apical brush border membrane of intestinal epithelial cells [29]
	large intestine [28]	monolayer of epithelial cells [28]
Human	subcutaneous adipose tissues [30,31]	adipocyte and capillary plasma membranes [30], macrophages/immune cells [31]
	pancreas [32]	A cells, β cells [32]
	muscle [21]	the myofiber surface of type 1 and type 2 fibers [21]
	stomach body, pyloric antrum [33]	-
	ileum [34]	mucosal epithelium [34]
	colon [24]	the surface epithelium and upper half of the crypt epithelium [24]
